# Validity and Reliability of Force Insoles to Measure Center of Pressure During Return-to-Sport Testing

**DOI:** 10.3390/s26010066

**Published:** 2025-12-22

**Authors:** Delaney McNeese, Charles Eisner, Rachel Todd, Brian Noehren, Meredith K. Owen

**Affiliations:** 1 Department of Biomedical Engineering, University of Kentucky, Lexington, KY 40536, USA; dmmc240@uky.edu; 2 Department of Biosystems Engineering, University of Kentucky, Lexington, KY 40536, USA; charles.eisner@uky.edu; 3 Department of Exercise Sciences, Brigham Young University, Provo, UT 84602, USA; rbt03@byu.edu; 4 Department of Physical Therapy, University of Kentucky, Lexington, KY 40536, USA; mow229@uky.edu

**Keywords:** center of pressure, force insoles, reliability, validity, return to sport

## Abstract

Center of pressure is a valuable biomechanical variable, predicting joint loading contributions during movement and giving insight into compensatory patterns. The purpose of this study was to assess the validity and reliability of force insoles in calculating vertical ground reaction force and center of pressure during return-to-sport jump testing. Ten healthy individuals performed double- and single-leg vertical and horizontal jumps on an instrumented treadmill while wearing instrumented force insoles. Vertical ground reaction force and anterior–posterior and medial–lateral center of pressure were collected at peak vertical ground reaction force from both devices. Repeat testing occurred 7 ± 5 days following the initial session. Force insoles were valid for measuring vertical ground reaction force (mean absolute error (MAE): 4.34 N/kg) and anterior–posterior center of pressure (MAE: 10% foot length) but were not valid for medial–lateral center of pressure (MAE: 50% foot width). During double-leg vertical, single-leg vertical, double-leg horizontal, and single-leg horizontal jumps, force insoles demonstrated good reliability for measurements of vertical ground reaction force (ICC: 0.89, 0.75, 0.89, and 0.91), anterior–posterior center of pressure (ICC: 0.88, 0.89, 0.94, and 0.97), and medial–lateral center of pressure (ICC: 0.72, 0.09, 0.82, and 0.73). Force insoles are a valid and reliable alternative to evaluating vertical ground reaction force and anterior–posterior center of pressure during return-to-sport jump testing.

## 1. Introduction

Center of pressure (COP) is a valuable biomechanical variable across a range of sports and orthopedic injuries, providing insights into compensatory joint contributions during a movement. The center of pressure is the location of the vertical ground reaction force (vGRF) during movement, and the ground reaction force vector is a major determinant of all lower extremity joint loading. For example, when COP shifts anteriorly, the ground reaction force moves further from the ankle and hip joint centers, increasing their external flexor moment arms, while moving closer to the knee, thereby decreasing its external flexor moment arm [1]. Manipulation of the COP reflects a compensatory strategy that redistributes joint loading when strength is asymmetric or post-injury. Furthermore, COP has been shown to predict up to 70% of between-limb differences in the knee extensor moment post anterior cruciate ligament reconstruction (ACLR) [2] and is associated with Achilles tendon loading [3], patellofemoral joint loading [4], and osteoarthritic movement patterns [5].

In addition to research applications in joint loading, COP is clinically insightful. Return-to-sport readiness is often measured by vertical or horizontal jump tests, with success measured by symmetry in distance to the uninvolved limb, while joint mechanics and loading symmetry are overlooked [6]. Physical therapists can consistently detect kinematic differences starting at 12°, so quantitative tools are necessary to measure smaller differences that go unnoticed [7]. Additionally, in some cases, kinematics may be symmetrical, while kinetic asymmetries persist, such as differences in vGRF magnitude and COP [8], leading to incorrect assumptions of recovery. COP measurement offers a way to quantify both kinematic and kinetic asymmetries. Both AP and ML COP can detect inter- and intra-limb joint loading compensations, which are valuable to assess return-to-sport readiness [2,9]. However, COP is most commonly assessed with force plates, which can be expensive and provide only a stationary area of assessment (i.e., small, usually square or rectangular). In order for COP to be a practical outcome for clinicians to assess, a more accessible solution to measure COP is required [10].

Force-instrumented insoles may provide a more clinically accessible tool for the assessment of vertical ground reaction force and COP measurement. However, before being used clinically, it is important to first establish the validity and reliability of COP measurement using force insoles. While prior research has quantified the COP validity and reliability of more controlled movements [11,12], no work has evaluated the validity and reliability of force insoles for return-to-sport jump testing. Thus, the purpose of this study was to assess the validity and reliability of force insoles in calculating vertical ground reaction force and center of pressure during return-to-sport jump testing.

## 2. Materials and Methods

To evaluate the validity and reliability of force insoles, healthy, active participants, defined as having no history of lower extremity injury, were recruited for this Institutional Review Board (University of Kentucky no. 79829)-approved study. The study followed established ethical standards laid out by the Declaration of Helsinki. Before completing any study-related activities, participants provided written informed consent. Participants completed two identical data collections approximately one week apart.

Standard three-dimensional motion capture was collected during return-to-sport jump testing to calculate ML and AP COP in the coordinate system of the foot. Participants had 25 retroreflective markers applied, with 17 placed across lower extremity anatomical locations and 2 rigid clusters of 4 markers each tracking the right shank and thigh segments throughout motion. Anatomical markers were placed on the right metatarsals, malleoli, tibial and femoral condyles, and the left and right greater trochanters. Four return-to-sport jump variations were assessed in the listed order: double-leg countermovement jump (DL CMJ), single-leg countermovement jump (SL CMJ), double-leg horizontal jump (DL HJ), and single-leg horizontal jump (SL HJ) [13]. For vertical jumps, prior to data collection, participants performed a maximal effort jump for height to experience what their maximal jump height felt like. Participants then completed four vertical jumps at 50% effort. Submaximal effort was assessed to focus analysis on the reliability of the force insole, rather than the participants’ ability to perform a consistent maximal effort jump from visit to visit. Participants also performed maximal effort double- and single-leg horizontal jumps to establish baselines. Jump distances were measured and recorded to scale trial jumps to 50% of maximum distance. To do this, markers were placed next to the force plates to identify the start and target end positions for horizontal jumps. Horizontal hop distance was visually confirmed using the target markers placed by the treadmill, and a jump was repeated if it landed outside the target window. A jump trial was considered successful based on visual observation if the participant maintained control of their center of mass and did not move their feet after the initial landing. Data were collected using a 13-camera motion capture system (Motion Analysis Corp, Rohnert Park, CA, USA) sampling at 200 Hz, with ground reaction forces recorded on an instrumented force plate (Bertec Corporation, Columbus, OH, USA) sampling at 2000 Hz [14,15]. Marker trajectories were filtered using a 4th-order low-pass Butterworth filter at 8 Hz. AP and ML center of pressure were calculated using Visual3D (C-Motion, Germantown, MD, USA) using the built-in COP Path function. AP and ML COP were output in meters.

Data were simultaneously collected at 100 Hz using instrumented force insoles (Moticon, Munich, Germany). These insoles were selected for their ability to directly calculate the center of pressure. Moticon OpenGo force insoles have 9 different sizes, and we used sizes S4–S9 across participants corresponding to shoe sizes 7 W–13 M. Each insole has 16 pressure sensors covering ~65% of the insole. There are 6 pressure sensors in the anterior–posterior direction and 2–5 in the medial–lateral direction. The insoles were calibrated according to Moticon’s recommended procedures [16]. The calibration includes slow walking, anterior–posterior shifts, and medial–lateral shifts. Moticon insoles output vertical GRF, AP COP, and ML COP.

Data were synced using a custom MATLAB code. Vertical ground reaction forces were down-sampled to 100 Hz to match the insole sampling frequency, focusing on the validity analysis of the force insoles within the constraints of their sampling rate. Down-sampling was performed by simple decimation. The indices of maximum vertical ground reaction force of each trial from both measurement systems were found. The data were then shifted by the difference between the two peak indices to align the data.

**Figure 1:**
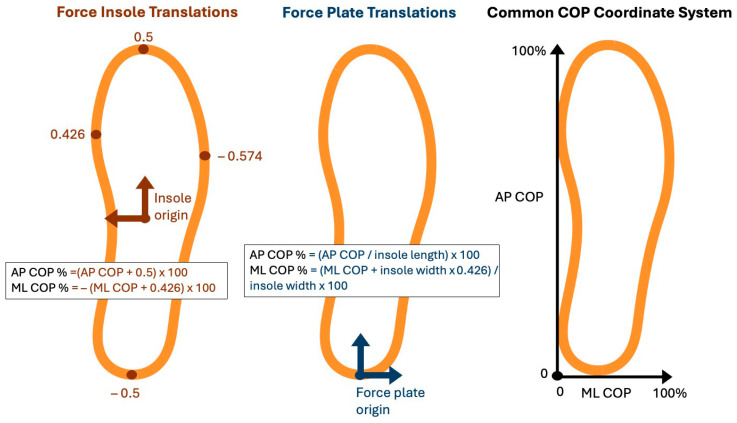
Anterior–posterior and medial–lateral centers of pressure were translated using the equations shown so that the origin became the most posterior, medial part of the foot. Anterior–posterior and medial–lateral center of pressure distances were scaled and are reported as a percentage of foot length and width, respectively. The data is reported relative to the scale on the right.

For comparison between measurement systems, both the force insole and force plate center of pressure data were translated into a common coordinate system, with the origin set to the most medial and posterior location of the foot, and represented as a percent of foot length/width (Figure 1). The origin of the raw insole AP COP was set to the center of the insole and on the scale of −0.5 to 0.5, with −0.5 representing the most posterior location of the foot and 0.5 representing the most anterior location of the foot. Insole AP COP measurements were translated from the local insole coordinate system to the common coordinate system and then multiplied by 100 to represent COP as a percentage of foot length (Equation (1)). Force plate AP COP was adjusted to be a percentage of insole length (Equation (2)). Raw insole ML COP was on the scale of −0.574 to 0.426, with −0.574 representing the most lateral aspect and 0.426 representing the most medial on the right insole. Insole ML COP measurements (Equation (3)) and force plate ML COP (Equation (4)) were translated from the local insole and force plate coordinate systems to the common coordinate system and reported as a percentage of insole width.

Insole AP COP % = (Insole AP COP + 0.5) × 100,(1)

Force plate AP COP % = (Force plate AP COP/insole length) × 100,(2)

Insole ML COP % = −(Insole ML COP + 0.426) × 100,(3)

Force plate ML COP % = ((Force plate ML COP + insole width × 0.426)/insole width) × 100,(4)

To assess validity, mean absolute error (MAE), root mean squared error (RMSE), and mean absolute percentage error (MAPE) were calculated for vGRF, AP COP, and ML COP outputs from force plates vs. force insoles. Pearson’s correlation coefficients (r) were also calculated to assess the linear relationship between the measurement systems. Validity was determined to be high if three of the following criteria were met: MAE and RSME less than 20% total magnitude, MAPE less than 20%, and Pearson’s r greater than 0.75. To assess reliability, intraclass correlation coefficients (ICC 3,k) using a two-way mixed effects model with absolute agreement were calculated between V1 and V2. Reliability was determined to be excellent if ICC was greater than 0.90, good if it was between 0.75 and 0.9, moderate if it was between 0.5 and 0.75, and poor if it was below 0.5 [17]. Each variable was analyzed at the time of peak vGRF during the movement.

## 3. Results

Ten individuals participated in the study. Full demographics and time between visits are displayed in Table 1.

**Figure 2:**
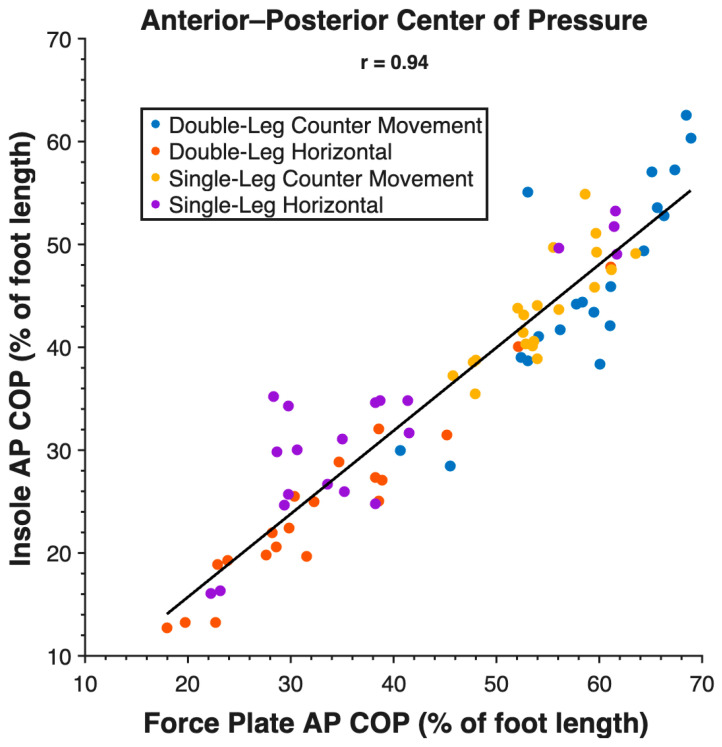
Anterior–posterior center of pressure (AP COP) measurements between force plates and force insoles during all tasks at peak vertical GRF.

**Figure 3:**
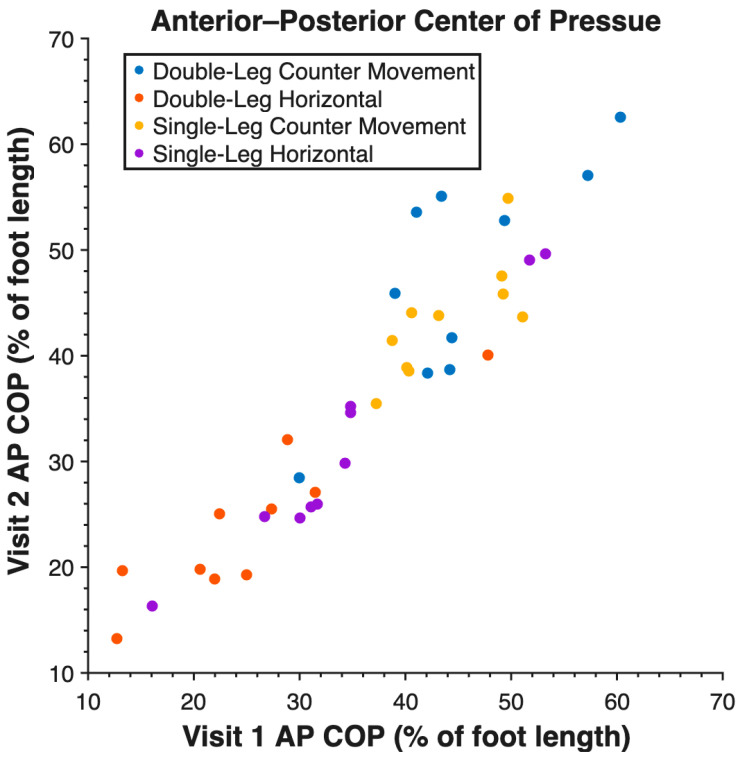
Anterior–posterior center of pressure (AP COP) of all tasks measured by force insoles between V1 and V2 at peak vertical ground reaction force.

**Figure 4:**
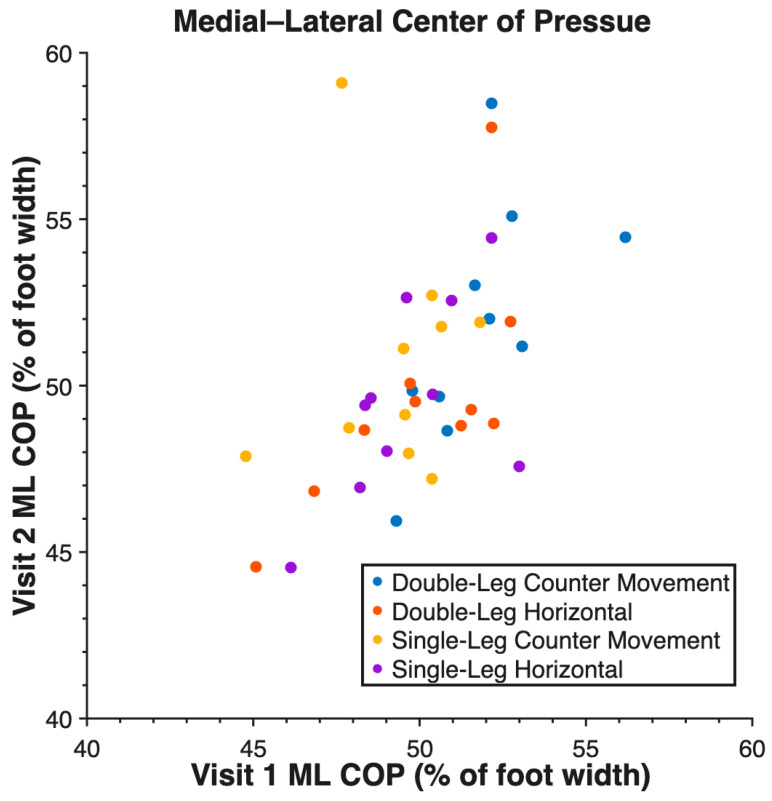
Medial–lateral center of pressure (ML COP) of all tasks measured by force insoles between V1 and V2 at peak vertical ground reaction force.

Validity measures are reported in Table 2. Force insoles demonstrated validity across all tasks for measuring vGRF and AP COP (Figure 2), but the ML COP measurement was not valid. Reliability measures are reported in Table 3. Reliability was high between visits for vGRF, AP COP (Figure 3), and ML COP (Figure 4).

Validity of the insole vGRF measurement is demonstrated by low MAEs across tasks (4.34 N/kg), low RMSE (5.68 N/kg), low MAPE for vertical jumps (9.21 N/kg and 9.72 N/kg), moderate MAPE for horizontal jumps (21.55 N/kg and 19.63 N/kg), and strong Pearson’s r across tasks (0.79). Validity of the insole AP COP measurement is demonstrated by low MAEs across tasks (10% of foot length), low RMSE (11% foot length), moderate MAPE (21.41% foot length), and a strong Pearson’s r across tasks (0.94). The insole ML COP measurement did not demonstrate validity through high MAEs across tasks (50% of foot width), high RMSE (50% foot width), and a weak Pearson’s r across tasks (0.20). Additional figures with task-specific correlations are presented in Appendix A.

## 4. Discussion

This study assesses the validity and reliability of force insoles for calculating vertical ground reaction force (vGRF) and center of pressure during return-to-sport jump testing. Force insoles demonstrated validity for vGRF and anterior–posterior center of pressure (AP COP) but were not valid for medial–lateral center of pressure (ML COP). Force insoles demonstrated high reliability for vGRF, AP COP, and ML COP for all tasks. These results suggest that force insoles can provide clinically meaningful measurements of vGRF and AP COP at peak vGRF.

Peak vGRF error magnitudes were moderate (MAE = 4.34 N/kg, RMSE = 5.68 N/kg, MAPE = 15.03%), while correlations were strong (r = 0.79). This pattern indicates that the force insoles inflated peak magnitudes relative to the force plates. This slight overestimation may result from the way insoles compute force (pressure × sensor area) compared to direct force measurement from strain gauges in force plates. The strong correlations suggest that while the absolute magnitude may be biased, the pattern of loading across participants and tasks was preserved, making insole vGRF values useful for relative comparisons.

Force insole AP COP measurements were valid at peak vGRF. This suggests that force insoles are valid for detecting anterior–posterior COP shifts, which is clinically relevant for identifying compensatory joint loading strategies post ACLR [2,9]. By contrast, ML COP measurements were not valid at peak vGRF. This may reflect the smaller scale of foot width in the medial–lateral direction, combined with a quick movement. Changes in ML COP may need to be captured at a higher sampling frequency than 100 Hz or with a denser array of pressure sensors [18,19]. Prior work has established that a sampling frequency of 200 Hz provides more accurate bilateral landing data [18]. Additionally, the narrow regions of the foot had lower sensor density, being only two sensors wide on the distal half. This may contribute to the lower ML COP validity.

Reliability was high for vGRF, AP COP, and ML COP between visit 1 and visit 2. AP COP demonstrated the highest reliability with ICC values between 0.88 and 0.97. ML COP also demonstrated high reliability with valid ICC values between 0.72 and 0.82. There was one anomalous low ICC output of 0.09 for ML COP during a single-leg countermovement jump. The distribution of this data in such a small sample size, as shown in Figure 4, resulted in the low insole ML COP ICC.

Prior work in other conditions has quantified the validity and reliability of force insoles in calculating vGRF, AP COP, and ML COP during movements such as balance, walking, running, and sidestep cuts [11,12]. For example, Watkinson et al. found strong agreement between force insoles and force plates, demonstrating GRF validity during walking, with reduced agreement of COP metrics such as sway area during balance tasks [11]. Similarly, Morin et al. also compared force insoles to force plates and found higher accuracy with walking than running [12]. They also found that COP demonstrated higher accuracy in the AP direction than the ML direction when accounting for the percentage of foot length and width. In a clinical setting, AP COP may be the more meaningful direction, as it provides insight into sagittal plane loading patterns [1,2]. Together, these studies, along with the current study, emphasize that force insoles demonstrate high validity for vGRF and AP COP across a range of tasks, while ML COP is a less valid variable when calculated with force insoles.

This study was limited by the low sampling frequency (100 Hz) of the force insoles, which may not capture more variable measurements, such as ML COP. Another limitation is an individual’s ability to consistently reproduce 50% effort across sessions. However, we monitored participants’ effort during data collection, and the high ICC values suggest this added minimal error into the test–retest reliability analysis. An additional consideration is that the effect of deformation of the force insole and shoe on GRF and COP calculations is unknown. These factors should be considered when interpreting the validity and reliability of force insoles.

Future studies should investigate COP throughout a rehabilitation program to better understand how loading asymmetries persist and the relationship to inter- and intra-limb compensations. Development of a low-cost, portable, and intuitive program and interface would encourage clinicians to integrate COP assessments, identifying joint loading compensations that are not visually apparent.

Overall, force insoles provide a valid and reliable method to assess vertical ground reaction force and anterior–posterior center of pressure during return-to-sport jump testing. However, while the medial–lateral center of pressure is reliable, it is not valid during return-to-sport jump testing. Utilizing portable insoles allows for inter- and intra-limb compensatory patterns to be monitored during return-to-sport jump testing outside of a research environment while maintaining strong agreement with force plate measurements.

## Figures and Tables

**Table 1:** Subject demographics.

n (M:F)	10 (5:5)
Age (Average ± SD)	25.5 ± 3.80
Height (m) (Average ± SD)	1.74 ± 0.09
Mass (kg) (Average ± SD)	74.9 ± 12.4
Days between visits (Average ± SD)	7.2 ± 4.7

**Table 2:** Validity between vertical ground reaction force, anterior–posterior, and medial–lateral center of pressure measurements between force plates and force insoles during different tasks. Data were extracted at the peak vertical ground reaction force. V1 and V2 data were used.

Variable	Task	MAE (N/kg)	RMSE (N/kg)	MAPE (%)	r
GRF (N/kg)	DL CMJ	1.99	2.50	9.21	0.84
	SL CMJ	2.94	4.15	9.72	0.76
	DL HJ	6.19	7.28	21.55	0.94
	SL HJ	6.22	7.25	19.63	0.68
	All Tasks	4.34	5.68	15.03	0.79
		MAE (%)	RMSE (%)	MAPE (%)	r
AP COP (%)	DL CMJ	13	14	22.37	0.85
	SL CMJ	11	11	19.85	0.83
	DL HJ	9	9	26.13	0.97
	SL HJ	6	7	17.29	0.91
	All Tasks	10	11	21.41	0.94
ML COP (%)	DL CMJ	49	49	*	0.25
	SL CMJ	54	54	*	0.73
	DL HJ	45	46	*	0.15
	SL HJ	51	51	*	−0.05
	All Tasks	50	50	*	0.20

* MAPE was not reported for ML COP because the scale of the variable resulted in the percentages being too high to interpret.

**Table 3:** Reliability, ICC (3,k), between V1 and V2 for vertical ground reaction force, anterior–posterior, and medial–lateral center of pressure measured by force insoles.

Variable	Task	ICC
GRF	DL CMJ	0.89
	SL CMJ	0.75
	DL HJ	0.89
	SL HJ	0.91
AP COP	DL CMJ	0.88
	SL CMJ	0.88
	DL HJ	0.94
	SL HJ	0.97
ML COP	DL CMJ	0.72
	SL CMJ	0.09
	DL HJ	0.82
	SL HJ	0.73

**Figure A1:**
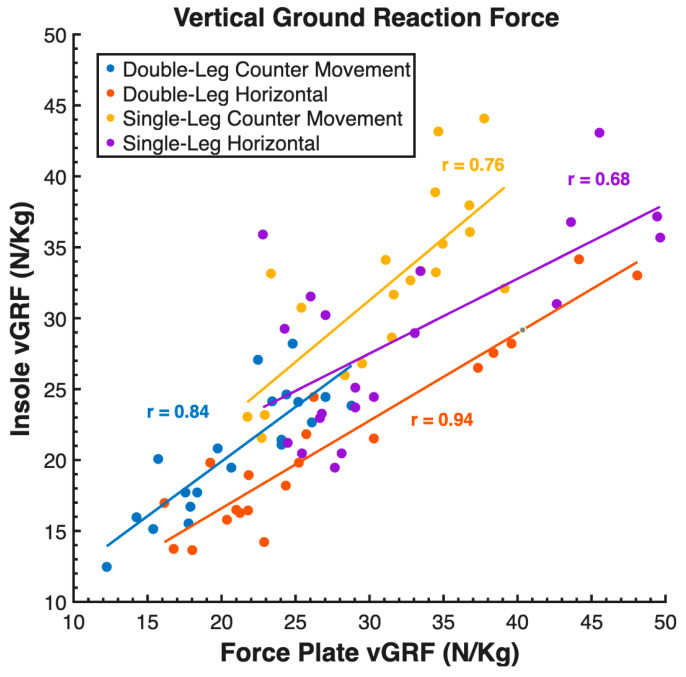
Vertical ground reaction force (vGRF) of all tasks measured by force insoles and force plate, with individual correlations plotted.

**Figure A2:**
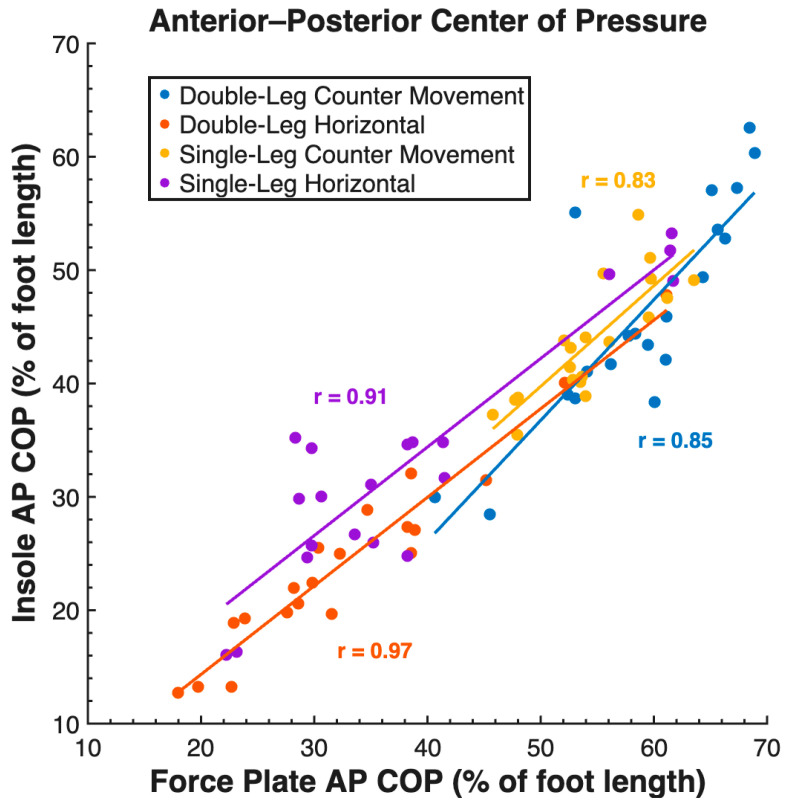
Anterior–posterior center of pressure (AP COP) of all tasks measured by force insoles and force plate, with individual correlations plotted.

**Figure A3:**
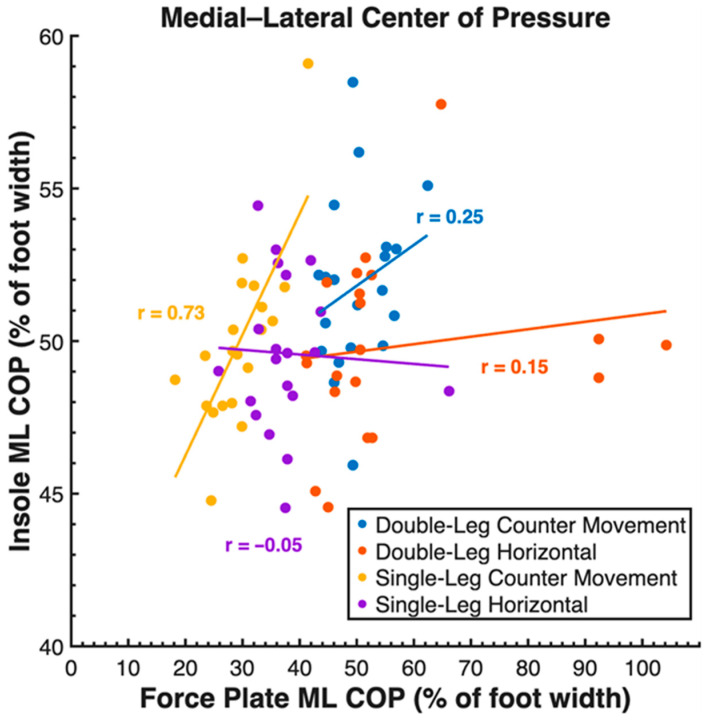
Medial–lateral center of pressure (ML COP) of all tasks measured by force insoles and force plate, with individual correlations plotted.

## Data Availability

The original contributions presented in this study are included in the article material. Further inquiries can be directed to the corresponding authors.

## Abbreviations

The following abbreviations are used in this manuscript:

COP	Center Of Pressure
AP COP	Anterior–Posterior Center of Pressure
ML COP	Medial–Lateral Center of Pressure
vGRF	Vertical Ground Reaction Force
DL CMJ	Double-Leg Countermovement Jump
SL CMJ	Single-Leg Countermovement Jump
DL HJ	Double-Leg Horizontal Jump
SL HJ	Single-Leg Horizontal Jump
MAE	Mean Absolute Error
RMSE	Root Mean Squared Error
MAPE	Mean Absolute Percentage Error
ACLR	Anterior Cruciate Ligament Reconstruction
